# MicroRNAs in Breastmilk and the Lactating Breast: Potential Immunoprotectors and Developmental Regulators for the Infant and the Mother

**DOI:** 10.3390/ijerph121113981

**Published:** 2015-10-30

**Authors:** Mohammed Alsaweed, Peter E. Hartmann, Donna T. Geddes, Foteini Kakulas

**Affiliations:** 1 School of Chemistry and Biochemistry, The University of Western Australia, Crawley 6009, Western Australia, Australia; E-Mails: mohammed.alsaweed@research.uwa.edu.au (M.A.); peter.hartmann@uwa.edu.au (P.E.H.); donna.geddes@uwa.edu.au (D.T.G.); 2 College of Applied Medical Sciences, Majmaah University, Almajmaah, Riyadh 11952, Saudi Arabia

**Keywords:** human milk, breastmilk, breastfeeding, RNA, microRNA, cells, lipids, skim milk, immune system, development, infant formula

## Abstract

Human milk (HM) is the optimal source of nutrition, protection and developmental programming for infants. It is species-specific and consists of various bioactive components, including microRNAs, small non-coding RNAs regulating gene expression at the post-transcriptional level. microRNAs are both intra- and extra-cellular and are present in body fluids of humans and animals. Of these body fluids, HM appears to be one of the richest sources of microRNA, which are highly conserved in its different fractions, with milk cells containing more microRNAs than milk lipids, followed by skim milk. Potential effects of exogenous food-derived microRNAs on gene expression have been demonstrated, together with the stability of milk-derived microRNAs in the gastrointestinal tract. Taken together, these strongly support the notion that milk microRNAs enter the systemic circulation of the HM fed infant and exert tissue-specific immunoprotective and developmental functions. This has initiated intensive research on the origin, fate and functional significance of milk microRNAs. Importantly, recent studies have provided evidence of endogenous synthesis of HM microRNA within the human lactating mammary epithelium. These findings will now form the basis for investigations of the role of microRNA in the epigenetic control of normal and aberrant mammary development, and particularly lactation performance.

## 1. Introduction

**Figure 1:**
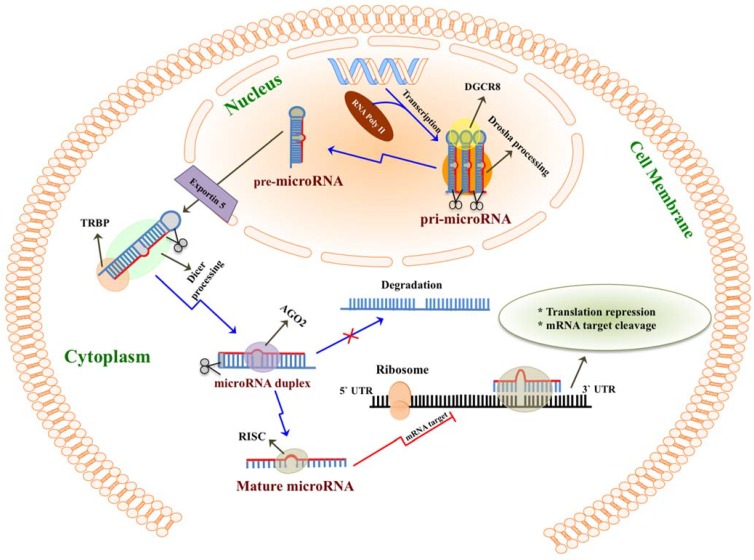
The predicted biogenesis of microRNA. MicroRNA are first transcribed from specific genes on DNA as primary microRNA (pri-microRNA) by RNA polymerase II (RNAPII). In the nucleus, pri-microRNA are converted into ~70-nucleotide precursor hairpin microRNA (pre-microRNA) by the enzymatic Drosha–DGCR8 complex. Pre-microRNA are then transported from nucleus to the cytoplasm by Exportin 5. There, the Dicer-TRBP complex produces ~20 base pair 3s′ microRNA and 5′ microRNA duplex. Dicer with assistance from argonaute 2 (AGO2) generates mature microRNA by cleaving the double strand of pre-microRNA. Only one strand of microRNA (3′ microRNA or 5′ microRNA) can be attached into the RNA-induced silencing complex (RISC). Finally, the microRNA/RISC complex binds into specific mRNA during protein translation, recognizing their target via a 6–8 nucleotides match-mir process (seed region). This results in either repression of the mRNA translation into protein or mRNA degradation.

Since their recent discovery in 1993, microRNAs (also known as miRNAs) have emerged as key regulators of gene expression at the post-transcriptional level in humans, animals and plants [1,2]. They act by binding to an mRNA target to either inhibit the translation of mRNA into protein and/or promote its degradation [3,4,5]. The microRNA family includes extremely small non-coding RNA (~22 nucleotide in length) that have been isolated from cells, tissues and body fluids of various mammalian species [6,7]. The biogenesis of microRNA comprises three main processes [8]: microRNAs are first transcribed into primary microRNA (pri-microRNA) from specific independent genes on DNA by RNA polymerase II, and are then converted into hairpin precursor microRNA (pre-microRNA) by the Drosha–DGCR8 complex. The enzyme Dicer then produces mature microRNA from pre-microRNA in the cytoplasm (Figure 1) [7,9]. According to miRBase version 21.0 (http://www.mirbase.org) released in June 2014, the number of pre-microRNAs in the human is estimated to be 1881. These correspond to 2588 known mature microRNAs, while the number of human protein-coding genes that are considered to be targets of microRNAs is estimated to be approximately 20,000–25,000 [10]. Therefore, a single mature microRNA can bind and regulate multiple mRNAs (genes) [6]. Importantly, ongoing research is still discovering new microRNAs.

In mammalian cells, various functional studies have demonstrated that microRNAs regulate up to 50% of protein synthesis (gene expression) [4]. Several roles of different microRNAs were investigated experimentally and they are involved in regulating a range of biological processes in plants and mammals (including humans) [3,11,12]. In addition to controlling normal physiological processes, microRNAs have been implicated in pathologies such as cancer, autoimmune diseases, gastrointestinal diseases, and diseases of the reproductive system [3,4]. microRNAs have recently been reported to be important regulators of pluripotency-related genes and they have been used to reprogram somatic cells into induced pluripotent stem cells (iPSCs) [13,14,15]. This regulation could potentially be an important method for regenerative medicine and biomedical research, as it eliminates the need for viral vectors. Viral vectors are used to reprogram cells into iPSCs, however they have been shown to uncontrollably influence reprogramming via random insertion of exogenous sequences into the genome [16,17].

Further to their role in the epigenetic regulation of stem cell fate and function, microRNAs also regulate the mammalian immune system. Their functions include regulation of T and B cell development [18,19], release of inflammatory mediators [20], proliferation of neutrophils and monocytes [21], and differentiation of dendritic cells and macrophages [22]. microRNAs are also thought to be involved in haematopoiesis [23], cardiac muscle development [24], insulin secretion [25], and neurogenesis [26]. Given their role in numerous physiological processes, deregulation of microRNA function can lead to disease; therefore, increasing evidence supports their use as diagnostic biomarkers. Either upregulation or downregulation of microRNAs has been found to be associated with initiation and progression of some types of cancers [27]. These include breast cancer [28], where upregulation of oncogene miR-2 has been shown to be involved in both initiation and progression of the disease [29].

High levels of microRNAs have been detected in body fluids, such as plasma, urine, saliva, seminal fluid, tears, cerebrospinal fluid (CSF) and more recently, milk [30]. Milk is a non-invasive source of numerous biomolecules that are either synthesized in the lactating breast or are transferred via the systemic circulation and provide important functions for both the lactating mother and the breastfed infant. To a large extent, milk microRNAs appear to be endogenous to the mammary gland [31] and could therefore be employed as biomarkers for both the performance and health status of the gland during lactation, and its aberrant growth associated with breast cancer. Further, food-derived microRNAs (e.g., exogenous miR-168a) [32] have been suggested to survive the mammalian gastrointestinal (GI) tract and regulate mammalian genes [32,33,34]. As human milk (HM, breastmilk) is highly enriched in microRNAs, it would be of great interest to illuminate the fate and function of this breastmilk component in the infant during breastfeeding and any long-term effects conferred during this period. Interestingly, bovine milk microRNAs miR-29b and miR-200c, which are also present in HM [35,36], have been shown to survive the GI tract of adult humans and increase in their serum post-consumption [37]. More recently, bovine milk exosomal microRNA transfer was demonstrated in human intestinal colon cells and rat small intestinal cells by endocytosis *in vitro* [38], further highlighting the important role of vehicle-mediated transfer of milk microRNA [39].

## 2. MicroRNAs Are Highly Enriched in Milk

### 2.1. microRNAs in Mammalian Milk

**Figure 2:**
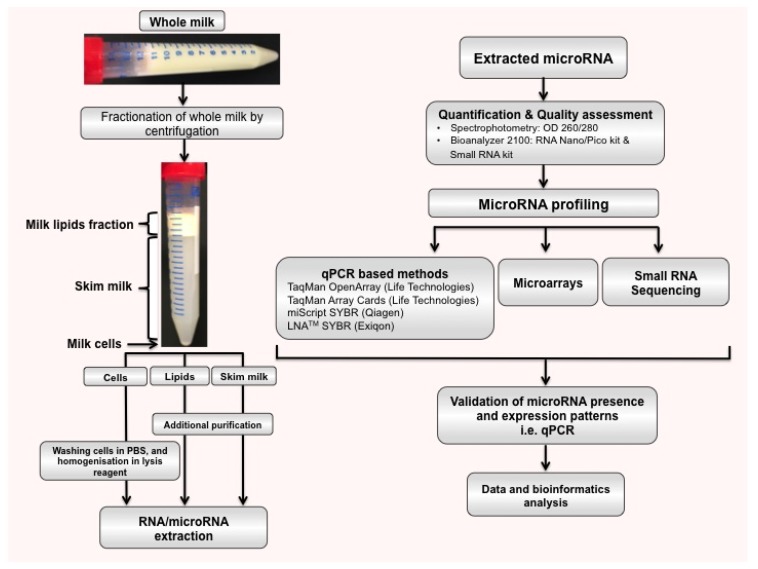
A workflow of microRNA identification in HM. Whole HM can be fractionated by centrifugation for 20 min at 800 g at 20 °C to obtain three fractions including the cells, the lipid layer and skim milk. Total RNA and microRNA can be extracted from each fraction using the optimal kit [47]. Profiling of microRNA after quantification and measurement of its quality can be performed using three different methods [47]: phenol/guanidine, filter column, and a combination of the filter column and phenol/guanidine methods. Small RNA sequencing can determine novel microRNAs and identify all microRNAs in a sample. Microarray analysis and qPCR-based methods can on principle only measure specific microRNAs. Validation of presence and expression patterns of a microRNA of interest is done using qPCR as it is highly sensitive and specific.

**Figure 3:**
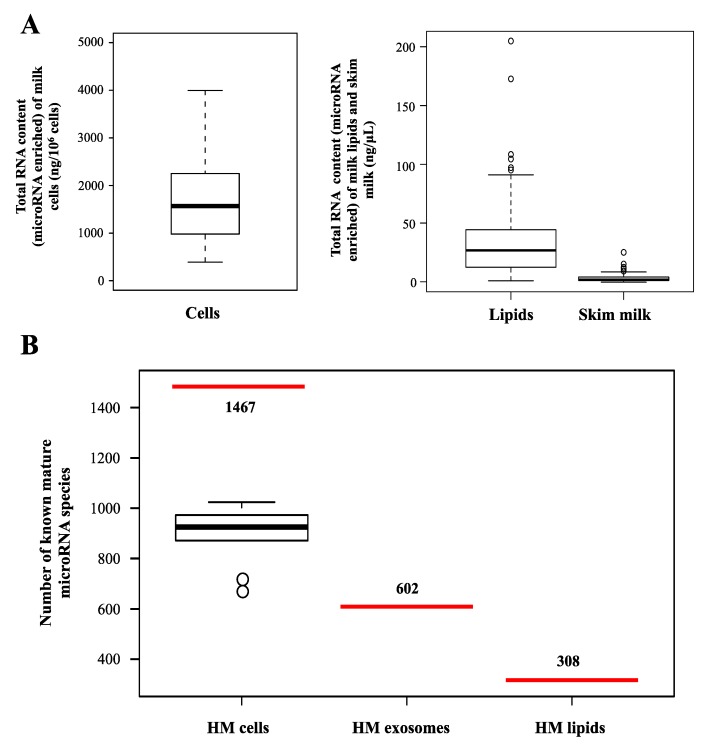
Differences in the microRNA content between the different fractions of human milk. (**A**) Box plots showing the total RNA content (enriched in microRNA) measured using NanoDrop 2000 in HM cells (*n* = 30 milk samples from 20 mothers), lipids (*n* = 127 milk samples from 79 mothers) and skim milk (*n* = 116 milk samples from 79 mothers) obtained from healthy breastfeeding mothers [35,45,47]. (**B**) Box plot showing the number of mature microRNA species that have been identified in HM cells, lipids and exosomes using deep small RNA sequencing. HM cell microRNAs were profiled in *n* = 20 samples collected from 10 healthy exclusively breastfeeding mothers in month 2 of lactation [35] using Illumina HiSeq2000, with total clean reads of 268,681,616 matched to miRBase version 20.0. This study identified 1467 different mature known microRNAs in HM cells. HM lipid samples (*n* = 7) were sequenced using an Illumina 1G Genome analyzer, with 124,110,646 clean reads mapped to miRBase version 16.0. This study identified 308 mature known microRNAs in HM lipids [44]. HM exosome samples (*n* = 4) were sequenced using an Illumina Genome analyzer II, with 83,520,000 clean reads matched to miRBase version 17.0. This study identified 602 mature known microRNAs in HM exosomes [36].

HM is considered the optimal food for term infants in the first six months of life [40], with the World Health Organization recommending exclusive breastfeeding for up to six months, with continuation of breastfeeding for at least the first two years [41]. In addition to providing nutrition, HM has long been known to protect the infant from infections and to play developmental functions integral to the infant, in which microRNAs are likely to be highly involved. microRNAs can be isolated and experimentally studied in the main three fractions of milk, the cells, lipids, and skim milk (Figure 2). Interestingly, HM is one of the richest microRNA source of all body fluids in the human, containing up to ~1400 mature microRNAs (Figure 3) [35,36,42,43,44,45,46]. Cellular and lipid fractions of HM contain a greater amount of microRNAs compared to the skim milk fraction [44,47], which is important to consider when analyzing milk microRNA. Not surprisingly, a wide variation in microRNA expression amongst lactating women has also been shown [48], with the factors that influence this variation having not been studied to date. Further, animal studies have shown that the type and expression levels of microRNA are distinctly different between the lactating and non-lactating mammary glands in the cow [49]. microRNAs were also found to be in involved in mammary gland development in murine models [50]. These animal studies have suggested a key role of microRNAs in the regulation of the development and performance of the lactating mammary gland, and they therefore have the potential to influence milk synthesis.

### 2.2. microRNAs in Different Milk Fractions

In 2010, Weber *et al.* isolated and profiled microRNA from 12 different human body fluids, including human colostrum and milk [30]. In the same year, microRNAs were profiled in skimmed HM [48]. Since then, a few studies have profiled microRNA in HM and in the milk of other mammalian species (Table 1) [36,44,48]. This was carried out using different platforms including qPCR, microarray analysis and small RNA sequencing [30,36,44,48,51,52]. Although qPCR is generally the method of choice providing high sensitivity and specificity, with low RNA input requirements, small RNA sequencing is often employed to screen for all microRNAs present. This allows identification of novel microRNAs and it is sensitive enough for microRNA quantification. Small RNA sequencing is done either using high throughput next generation sequencing (NGS) or small-scale NGS platforms [4].

Amongst the 12 human body fluids analysed, Weber *et al.* used qPCR to profile 429 known mature microRNAs in mature skimmed HM and 386 known mature microRNAs in skimmed human colostrum (the mammary secretion produced in the first few days postpartum) [30]. This study demonstrated for the first time the high abundance of microRNA in HM, which is in accordance with its high total RNA content compared to other body fluids (47,240 μg/L *vs.* 308 μg/L in plasma and 94 μg/L in urine) [30]. Microarrays were first used to profile microRNAs in HM by Kosaka *et al.*, who examined 281 different microRNAs in skimmed HM obtained in the first six months of lactation [48]. Although these two pioneering studies focused on microRNA analysis in skimmed HM, it has been argued that the other two main milk fractions (lipids and cells) are likely to harbor large quantities of microRNAs. Indeed, later Munch *et al.* analyzed the lipid fraction of HM using small RNA sequencing and found high quantities of microRNA, reporting 308 microRNA species that are predicted to target a total of 9074 genes [44]. In addition, a subsequent study performed deep sequencing to profile microRNAs from HM exosomes [36], which are small cell-derived vesicles carrying proteins and molecules present in all body fluids [53]. This study found 639 exosomal mature microRNAs originating from 452 pre-microRNAs [36].

**Table 1:** Number of mature known microRNA species reported for different mammalian species for the three milk fractions (cells, lipids and skim milk).

Species	Milk Fraction	microRNAs *	Profiling Method	Reference
Human	Skim milk (mature)	429	qPCR	[30]
	Skim milk (colostrum)	386	qPCR	[30]
	Skim milk (mature)	281	Microarray	[48]
	Milk exosomes	639 **	Solexa deep sequencing	[36]
	Milk lipids	308	Solexa deep sequencing	[44]
	Milk cells	450 ***	TaqMan OpenArray	[45,46]
	Milk lipids	337 ***	TaqMan OpenArray	[45,46]
	Milk cell pre-feed	1287	Solexa deep sequencing	[35]
	Milk cell post-feed	1308	Solexa deep sequencing	[35]
Bovine	Skim milk (colostrum)	230	Solexa deep sequencing	[52]
	Skim milk (mature)	213	Solexa deep sequencing	[52]
	Skim milk (colostrum)	100	Microarray	[54]
	Skim milk (mature)	53	Microarray	[54]
Porcine	Milk exosomes	180 **	Solexa deep sequencing	[51]
	Milk exosomes (colostrum)	491	Solexa deep sequencing	[55]
Murine (rat)	Skim milk (colostrum)	128	Microarray	[56]
	Skim milk (mature)	144	Microarray	[56]

* Number of detectable mature microRNAs; ** Precursor microRNAs (pre-microRNAs); *** 8 ≤ Ct ≤ 29.

More recently, we used the Taqman OpenArray Panel system (Applied Biosystems, Foster City, CA, USA) to screen 754 human mature microRNAs in the cellular and lipid fractions of HM. In addition, we performed comparisons with the microRNA content of maternal peripheral blood mononuclear cells (PBMCs) and plasma [46]. This analysis identified 293 and 233 microRNA species in the breastmilk cell and lipid fractions, receptively. Maternal PBMCs contained 345 different microRNAs, whereas only 169 were found in maternal plasma. Breastmilk cells and PBMCs had significantly higher microRNA content compared to breastmilk lipids and plasma, respectively (*p* < 0.05). Correlation and cluster analyses showed that breastmilk cells and lipids were highly related in terms of microRNA expression patterns and species, however PBMC microRNAs were not correlated with breastmilk microRNAs. In plasma, marked inter-individual variation in expression levels of single microRNA species was observed. This study together with our previously published optimization of microRNA extraction from HM [47] demonstrated the presence of microRNAs in all three fractions of HM (cells, lipids, and skim milk), and revealed that HM conserves more microRNAs with different expression patterns compared to maternal plasma [46], but also other human body fluids [30]. Skim milk (known as the plasma phase of milk) is cell and fat globule free milk, and appeared to be extremely low in microRNAs and total RNAs compared to the cell and lipid milk fractions [47].

In addition to HM, microRNA have been analyzed in other mammals’ milk, particularly the dairy cow, in various studies (Table 1) [51,52,53,54]. Mature bovine skim milk has been shown to contain 213 microRNA species using deep sequencing [52] and 53 using microarrays [54]. Some of these microRNA are enriched in either mature milk or colostrum [52]. Specifically, bovine colostrum has been found to be richer in microRNA (both higher total content and species number) compared to mature bovine milk, where 230 and 100 microRNAs were identified in skimmed bovine colostrum in the above studies, respectively (Table 1) [52,54]. Similarly, bovine milk-derived microvesicle RNA and total milk RNA levels have been shown to be higher in colostrum compared to mature milk [53,54]. Unlikely bovine milk, skimmed rat colostrum was shown to conserve fewer microRNA species (128) than mature rat skim milk (144) (Table 1) [56]. Izumi *et al.* [54] isolated 53 mature microRNAs from mature bovine skim milk, and 100 from skimmed bovine colostrum (Table 1). Gu *et al.* examined the exosomes of porcine milk using small RNA sequencing at six time points of lactation within the first month postpartum (0, 3, 7, 14, 21, and 28 days after birth) and found 180 pre-microRNAs encoding 237 mature microRNAs, which are also found in HM (Table 1) [51]. However, 39 pre-microRNA were identified that were not homologues of HM microRNA [51]. This was one of the first efforts to quantify changes in milk microRNA content over time, in this case within the first month of lactation, which can provide insight into both their involvement in the maturation of the mammary gland into an organ that synthesizes copious amounts of milk and their role(s) in the development and protection of the offspring. Another study in porcine milk exosomes identified 491 mature microRNAs by small RNA sequencing in the first 6 days postpartum (Table 1) [55]. These pre-microRNA that are shared between different species potentially have similar physio-pathological mechanisms of action and functions in milk amongst mammals [51]. In all animal milk microRNA studies to date, only a few microRNA were found to be highly expressed [30,36,44,48,51,52]. For example, the top 10 most highly expressed microRNAs in exosomal porcine milk were contributing approximately 87% of the total 234 microRNA [51]. 

### 2.3. Origin of Milk microRNAs

Our comparisons between breastmilk blood microRNA provided important insight into the origin of milk microRNA, with the mammary gland appearing to be the main source of milk microRNA, with the maternal circulation having a smaller contribution [46]. This was consistent with a recent microRNA analysis in tammar wallaby milk. In the tammar study, most microRNAs were differentially expressed between skim milk and blood serum, although the total number of microRNA species was similar in both milk and serum (86 and 82 microRNAs, respectively) [57]. Prior to these findings, it was believed that because ribosomal RNA (18S and 28S) measured by the Bioanalyzer is usually absent or low during the isolation and quantification of microRNA, microRNA are less likely to be secreted from milk cells [51,54]. Nevertheless, taken together, the similarities between milk cell and lipid microRNAs, the differences with PBMCs and plasma both in the human [45] and dairy cow [52], and the known secretion of milk lipids from lactocytes, which are the most abundant cell type in HM under healthy conditions [58], strongly suggest that the milk cell and lipid microRNAs are primarily endogenously synthesized in the mammary gland [45,57]. Moving forward, it will be important to understand the factors controlling mammary microRNA synthesis during pregnancy and lactation, as this is likely to impact the health and development of both the mammary gland and the infant.

### 2.4. Milk microRNAs as Diagnostic Tools

Although the microRNA content and composition of different milk fractions is being intensively investigated, the understanding of factors influencing them as well as the roles and functions of these molecules in the lactating mammary gland and for the breastfed offspring is still very poor. A number of maternal and/or infant characteristics have previously been reported to influence the composition of HM, including infant feeding, preterm birth, the stage of lactation, parity, maternal body mass index (BMI), infant sex, and the health status of the mother and the infant [59,60,61,62,63,64,65]. It is not unlikely that some of these factors may affect the microRNA content of HM, yet very few studies have examined these associations. Recently, the effects of infant feeding and milk removal on HM microRNA content and composition were investigated [45,46]. It is well established that post-feed milk contains more fat and cells compared to pre-feed milk [60,61]. Similarly, additional microRNA species were detected in post-feed milk, however the difference in the total number of microRNA species or the expression of the majority of microRNAs between pre- and post-feed milk was not statistically significant in a group of 10 lactating women examined in month 2 of lactation (*n* = 10, *p* > 0.05). Yet, a subgroup of 27 known and 1 novel microRNAs in this study were expressed more highly post-feeding (*p* < 0.05). From these findings, it can be concluded that milk removal may influence the content and/or expression of certain microRNAs in HM, but the overall microRNA composition appears to remain constant [35]. This is in agreement with Kosaka *et al.*, who showed variable microRNA expression patterns between mothers, but claimed no significant intra-individual variation in microRNA expression [48]. Yet, this study only collected 2–4 samples from each of eight lactating women at different stages of lactation without standardizing the sampling based on infant feeding/milk removal or time of the day. Therefore, further studies are required to shed light into factors that may influence HM microRNA content within a mother. Nevertheless, Kosaka *et al.* [48] as well as the more recent study by Alsaweed *et al.* [35] showed great inter-individual variation in HM microRNA content, which could potentially be associated with parity, preterm birth, infant characteristics or environmental factors (e.g., maternal diet) [37,66]. Interestingly, maternal diet has been shown to influence other HM components, such as fatty acids [67] during lactation, but also fetal growth and health during pregnancy [68,69].

Similarly to numerous other immunological components of HM (immune cells, lysozyme, lactoferrin, immunoglobulins [62,70]), immune-related milk microRNA may be influenced by the health status of the mother and/or the infant. Although this has not been investigated in HM, a recent study in the dairy cow examined milk from healthy cows and those infected with *S. uberis* 0140J. It was found that 26 microRNAs isolated from milk cells described as monocytes were differentially expressed between the two cohorts [71]. The majority of the differentially expressed microRNAs are implicated in innate immunity, suggesting that infection of the lactating breast changes the milk microRNA profile to enhance immunoprotection and facilitate recovery. It is of note that in this study, milk monocytes were identified using the marker CD14, which is also expressed by milk epithelial cells [58,70]. Although epithelial cells are not the dominant cell type in bovine milk, in contrast to HM [58,70], the microRNA profiles reported in this study are likely to represent more cell types than just monocytic immune cells of milk.

Although in HM the effects of infection on microRNA profiles are currently under investigation, evidence from animal studies supports the use of milk microRNA as a tool of assessing the health status of the lactating breast as well as the response to treatment, similarly to what has been previously shown for breastmilk immune cells [62,70]. The potential diagnostic value of milk microRNA has also been suggested by other animal studies. miR-148a-3p, which has been found to be the most highly expressed microRNA in exosomes of HM [36], bovine [52] and porcine milk [51], has been proposed as a biomarker for raw milk quality control in the dairy industry, and also for artificial infant formulae [52]. The use of microRNAs as biomarkers for milk quality control was first proposed by Chen *et al.* due to their high stability in milk, even under very harsh conditions including the sterilizing process during product manufacture and milk processing [52]. However, Weber *et al.* [30] reported a lower concentration of miR-148a in skimmed HM than what was previously shown in bovine skimmed milk by Chen *et al.*, in HM exosomes by Zhou *et al.*, and in porcine milk exosomes by Gu *et al.* [36,51,52]. In addition to miR-148-3p, controversies exist over miR-494, which has been identified to be present in high concentrations in both HM [48] and bovine milk by Izumi *et al.* [54], but in very low concentrations in bovine milk by Chen *et al.* [52]. It is not clear the degree to which inter- and intra-species variations and factors associated with them have contributed to these differences. Further, differing methodological approaches and lack of standardization of milk collection, storage, processing, milk fractionation (if any), and RNA extraction are also likely sources of potential variation in results. Due to the rapidly evolving techniques in this field, the need is arising for greater emphasis of studies to optimize and standardize the methodology employed in milk microRNA research. These procedures had already been optimized for microRNA extraction and analysis in blood and plasma [72], and only recently this has been done for HM [47].

The optimization of microRNA and total RNA extraction from HM was conducted in three main milk fractions (cell, lipids and skim milk) using different extraction methods and commercially available kits. The most efficient kits and methods were reported for each HM fraction [47]. In this study, microRNAs were found to be enriched in HM, with different milk fractions yielding different microRNA concentrations [45]. Therefore, it became clear that different fractions of HM require different processing for extraction, profiling and functional studies. Importantly, milk samples were fractionated and analysed fresh upon expression and not after storage, enabling extraction and analysis of microRNA specific to each milk fraction. Previous studies on skim milk or milk lipids have typically analysed milk after freezing [36,48,51], a process that is likely to result in cross-contamination between milk fractions due to membrane lysis (in milk cells and potentially fat globules) that is known to occur during freezing. These are important considerations for future investigations, with this optimization study now providing a standard protocol for HM microRNA analysis [47].

Furthermore, the optimisation and standardization of the methodology for milk microRNA analyses [47] opens new avenues for clinical exploitation of these molecules diagnostically, particularly given their non-invasive access via breastmilk. The suggested origin of many milk microRNA from the mammary gland [45,57] makes them an attractive target as biomarkers of the health status and performance of the lactating breast as well as of breast aberrations such as cancer. Epigenetic modification has been suggested to be involved in the normal development of the mammary gland, although the specific mechanisms are still largely unexplored [73]. For example, miR-29s was found to regulate important lactation-related genes in mammary epithelial cells from the dairy cow, such as casein alpha S1 (CSN1S1), E74-like factor 5 (ElF5), and glucose transporter 1 (GLUT1) [52,74]. Decreasing expression of miR-29s was associated with reduction of lactoprotein, triglycerides (TG) and lactose [52,74]. These findings can form the basis for examination of potential avenues for enhancement and optimisation of milk quality in the dairy cow, as well as improvement of lactation performance in women with insufficient milk supply.

## 3. Functions of Milk microRNAs

### 3.1. Stability and Uptake of Food-Derived microRNAs

Accumulating evidence confirms that microRNAs are present in all food sources. A number of studies have begun to investigate the fate of food-derived microRNAs, and whether they survive the GI tract and influence gene expression in mammals, including humans [32,33,34,37]. Food-specific microRNAs ingested orally have been found to be present in tissues and sera of different animals. Specifically, exogenous miR-168a, which is a rice-specific microRNA, was present in human sera in a Chinese cohort [32]. miR-168a was found to bind low-density lipoprotein receptor adapter protein 1 (LDLRAP1) in the human and mouse and to inhibit LDLRAP1 expression in the liver [32], demonstrating not only survival and uptake of this food-derived microRNA in humans, but also epigenetic regulation influencing tissue function. However, not all cross-microRNAs from food sources increase after consumption in mammals. For example, miR-167a and miR-824 are highly expressed in broccoli. Extensive consumption of broccoli sprouts by healthy humans did not change the expression pattern of either microRNA in the plasma [37]. Moreover, Dickinson *et al.* investigated exogenous microRNA uptake in mice by feeding them rice-containing chow. This study failed to report gene-targeting functions of the plant-derived microRNA or change in expression levels in the liver or plasma of the animals [75]. The above discrepancies between studies examining plant-derived microRNA transfer to mammals may reflect the lack and/or minimal contribution of exosomal transfer, since very limited evidence currently supports the production of exosome-like structures by plants that can be uptaken by mammalian cells [76]. Indeed, the packaging of microRNA within “transporting vehicles” may play an important role for their transfer and function in the recipient.

**Figure 4:**
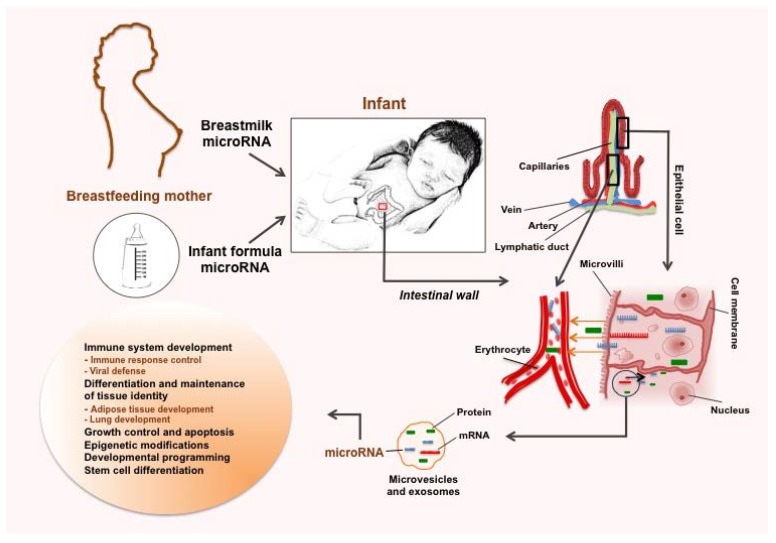
A potential scenario depicting the sources of exogenous microRNA for the infant (breastmilk and infant formulae) and uptake of them along with other macro/micronutrients (*i.e.*, fatty acids and amino acids) in the infant’s gastrointestinal (GI) tract. Breastmilk microRNAs can be delivered to the infant either as free molecules in skim milk, or via uptake of breastmilk cells, exosomes and other milk microvesicles in the GI tract. There, absorption is thought to occur through intestinal epithelial cells, from which milk-derived microRNA may reach various organs and tissues via the bloodstream to potentially perform functions, such as immunoprotection and developmental programming. It is of note that infant formulae are extremely poor in microRNA compared to HM, with potential differences also in the biological activity of these molecules in formula that merit further investigation.

In the case of HM microRNA, it has been suggested that their transfer to the infant’s bloodstream is further facilitated by the known packaging of milk microRNA in “vehicle” structures, such as somatic cells, exosomes and other microvesicles, which may be essential for the long-distance transport of microRNA, given that they are surrounded by a lipid bi-layered membrane and are equipped with adherence molecules, both of which facilitate their ordered endosomal transfer via epithelial cells of the intestine [38]. Through these vehicles, milk-derived microRNA are thought to be uptaken by the infant and participate in the epigenetic regulation of various functions including immune protection and development (Figure 4) [34,77]. In particular, recent studies have emphasized the importance of exosomal transfer of milk-derived microRNA. Extracellular vesicles including exosomes were shown to attach to different types of cells by endocytosis and to carry microRNA such as miR-21, which downregulated expression of TGFβRII and TPM1 in the recipient cells [78,79,80]. Extracellular vesicles were further investigated in commercial bovine milk, and were found to carry immunoregulatory microRNAs. These milk-derived extracellular vesicles were resistant to harsh conditions such as low pH [39]. Moreover, uptake and functionality, including therapeutic effects, of milk microRNAs has been recently demonstrated both *in vitro* and *in vivo* by Arntz *et al*. [81]. In this study, immune-related microRNAs (miR-30a, miR-223, miR-92a) were highly expressed in bovine milk-derived extracellular vesicles, which were uptaken *in vitro* by splenocytes and intestinal cells. When orally administered to BALB/c mice with experimental rheumatoid arthritis, these milk-derived extracellular vesicles were uptaken by RAW264.7 macrophages after 1–3 h as well as by the ileum tissue of the animals after 24 h. After 9 weeks of daily oral administration, arthritis was delayed, with reduction in cartilage depletion and joint inflammation [81].

In addition to exosomal transfer, HM cells may significantly contribute to the transfer of milk-derived microRNA to the infant. It has been recently shown that stem cells and immune cells from milk are transferred to the bloodstream of suckling pups in mice, and from there to different tissues [82,83]. Given that the cells of milk are highly rich in microRNA [35,45], this is likely to be an important source of microRNA for neonates in addition to milk exosomes.

microRNAs contained in infant formulae may also, to a small extent, be transferred to the infant’s circulation. Baier *et al.* investigated bovine milk-derived miR-29b and miR-200c in human adults after consuming cow’s milk and found that both microRNAs were increased 2-fold in human PBMCs and could potentially alter gene expression [37]. Both microRNAs were also highly expressed in the human plasma after few hours of consuming cow’s milk, and returned to the normal baseline expression level after 24 h of the initial milk consumption [37]. Furthermore, bovine milk exosomes isolated from commercial milk products were shown to be transported into human intestinal colon carcinoma Caco-2 cells and rat primary small intestinal IEC-6 cells by endocytosis *in vitro*, a process that was influenced by glycoproteins on the surface of host cells [38]. However, differences exist in the microRNA content between bovine milk and infant formula, with the latter lacking exosomes and viable cells, and thus containing much lower microRNA concentrations (approximately 100-fold lower in bovine milk-based formula compared to raw bovine milk and colostrum [52,84]) [45]. Moreover, the non-human origin of formula microRNA and/or the procedures of formula preparation may be associated with altered biological activity of any remaining microRNA in infant formula.

One of the major requirements for confirmation of the functionality of food-derived microRNAs in mammals is to demonstrate their survival in the GI tract. Several studies have shown that microRNAs are extremely stable under various harsh conditions *in vitro* [36,48,85]. For breastmilk microRNA, the main considerations are resistance to RNase digestion and tolerance of low pH, and temperature and freeze/thaw cycles in the case of frozen HM [36,48,53]. Exosomal microRNA has been suggested to be protected [36], but other microvesicles including fat globules are also considered to be involved in microRNA protection, such as apoptotic bodies (small vesicles derived from apoptotic cell death) [48], which have not been investigated yet. Moreover as stated above, milk cellular microRNA may be transferred intact as it is protected within cells, which have been shown to survive the GI tract of the offspring and to home in different organs [32,57].

With regard to free microRNA in milk (such as those present in skim milk), a few theories have been proposed. Ribonuclease (RNase), which has been found to exist in all body fluids [86], degrades RNA molecules into small fragments, and is thus a key enzyme in the RNA maturation process [87]. Milk is known to have high RNase activities [36,48]. On the other hand, it is known that RNAs are unstable under harsh conditions [88,89]. Furthermore, HM and raw milk-derived microRNAs are found to be extremely stable even after RNase treatment *in vitro* [48,51,53,54]. The effects of low pH solution on microRNA integrity were examined using qPCR, showing that they are very stable [48,51]. It is important to note that the GI tract of infants is less acidic than that of adults [90], which further supports increased survival of milk microRNA activity. Moreover, milk microRNAs are resistant to milk storage under different temperatures, such as incubation at 100 °C for 10 min, and freeze-thaw cycles [36,48,51]. As microRNA do not denature if subjected to different temperature cycles (at least those tested), microRNAs in stored HM fed to hospitalized infants are likely to be unaffected [48]. The above observations strongly support the survival of the natural microRNA content of HM in the infant’s GI tract, either as free molecules or packaged in vesicles/cells, and thus suggest a potential function of these transferable and stable molecules in the breastfed infant, including the hospitalized infant receiving stored HM.

A recent study utilized a mouse model of miR-375 and miR-200c knockout (KO) pups fed by wildtype (WT) foster mothers or KO mothers [91]. The study concluded that no evidence was found for intestinal uptake in KO or WT pups of miR-375 and miR-200c derived from foster mother milk [91]. However, a small increase in the plasma levels of both of these microRNAs was detected in KO pups after nursing, suggesting that some microRNA copies are actually transferred to the bloodstream. It is of note that the examined microRNAs were not highly expressed in the WT mother milk of the murine model used. Further, both miR-375 and miR-200c are known to be involved in the control of endocytosis and/or exocytosis and to modulate epithelial function, which may influence exosomal endocytosis and thus uptake of the examined microRNAs [92,93]. Therefore, this KO mouse model and the chosen microRNAs may be inappropriate for investigating milk microRNA uptake by the nursed offspring. As it has been previously shown [37,38], not all dietary microRNAs are ideal for exogenous microRNA uptake studies [94]. Further studies are required to confirm the findings of Title *et al.* [91] as well as investigate milk microRNA uptake in more appropriate models.

On the other hand, similar to human serum [89], exogenous spiked-in synthesized (artificial) microRNAs in bovine milk were unstable and degraded compared to natural, endogenous microRNAs in bovine milk, which remained stable [51,54]. Interestingly, it is known that HM contains high quantities of very stable microRNAs, which are resistant to the pasteurization and milk bank storage procedures [36,48]. Additionally, microRNAs were found to be active and still regulate their target genes after subjection to ultraviolet radiation (UV-A, UV-B, and UV-C) [95]. In *C. elegans*, several microRNAs were stably expressed after UV-C treatment, such as miR-57-5p and miR-55-3p [96]. In a study of human primary keratinocytes exposed to UV-A and UV-B for 6 h, most microRNAs survived and no difference in expression was seen, except for few microRNAs such as miR-23b (upregulated) and miR-10a (downregulated) [97]. Yet, other studies have reported upregulation of skin microRNA in response to UV irradiation [98], which may be involved in cancer initiation in the skin [99]. In summary, although ultraviolent radiation may not affect the majority of microRNA in mammalian cells, current evidence suggests that some microRNA species may be affected, and therefore this requires further investigation. In general, as it is also emphasized by the food industry and the industry of processing raw milk products such as milk powdechen [52,54], microRNAs in food and milk are very stable under and resistant to harsh conditions, and are therefore likely to be taken up by the HM fed infant, especially when protected within vehicle structures such as exosomes and live milk cells.

### 3.2. microRNAs Act as Immune Regulators

The tolerance of microRNAs of harsh conditions and the evidence that they migrate to the bloodstream and potentially different organs of the breastfed infant, suggest that they may play functional roles in the epigenetic regulation of development. Most of the microRNAs in HM are known for their immunocompetence [54,100,101], and they are particularly abundant (Table 2) [30,36,44,48]. They are thought to be involved in several mechanisms of the immune system, such as regulation of B and T cell differentiation and development, and innate/adaptive immune responses [100,101]. In addition, microRNA can play key roles in autoimmune conditions, such as inflammatory bowel disease (IBD), and regulate the development or prevention of these diseases [102]. Therefore, they could potentially be used as milk biomarkers to diagnose immune disorders such as allergic conditions [103,104].

Kosaka *et al.* [48] reported high quantities of microRNAs in HM with functions associated with the immune system during the first 6 months of lactation, including, but not limited to miR-181a, miR-17, miR-155, miR-150, and miR-223. In particular, miR-181 and miR-155, which are known to regulate B cell differentiation [48,105,106], are present in high concentrations in HM [44,48], suggesting a function in the development of the infant’s immune system.

**Table 2:** Immune-related microRNAs expressed in different fractions of milk from different mammalian species that have been highlighted in previous studies.

MicroRNA	Regulatory Function(s)	References	Presence in HM	References	Presence in Animal Milk	References
miR-181a	Cell signaling. Development of B cells.	[105,107]	Skim milk. Milk lipids.	[30,44,48]	Bovine skim milk (colostrum and mature milk). Rat milk whey. Porcine milk exosomes.	[52,55,56]
miR-181b	Switch recombination in activated B cells. Increased activity of NF-κB.	[105,108,109]	Skim milk (colostrum and mature milk). Milk lipids.	[30,44,48]	Bovine skim milk (colostrum and mature milk). Rat milk whey. Porcine milk exosomes.	[52,55,56]
miR-155	B and T cell differentiation. Innate/adaptive immune response.	[106,110,111]	Skim milk (colostrum and mature milk). Milk lipids. Milk exosomes.	[30,36,44,48]	Bovine skim milk (colostrum and mature milk).	[52,54]
miR-17	B and T cells. Monocyte development.	[112,113]	Skim milk. Milk lipids. Milk exosomes.	[36,44,48]	Bovine skim milk (colostrum and mature milk). Rat milk whey. Porcine milk exosomes.	[52,55,56]
miR-92a	B and T cells. Monocyte development. Downregulated in lymphoma.	[114,115]	Skim milk (colostrum and mature milk). Milk lipids.	[30,44,48]	Bovine skim milk (colostrum and mature milk). Rat milk whey. Porcine milk exosomes.	[52,54,55,56]
miR-125b	Tumor necrosis factor-α production. Innate immune response. TLR signaling.	[111]	Skim milk (colostrum and mature milk). Milk lipids.	[30,44,48]	Bovine skim milk (colostrum and mature milk). Rat milk whey. Porcine milk exosomes.	[52,54,55,56]
miR-146a	Innate immune response. TLR signaling.	[116,117]	Skim milk (colostrum and mature milk). Milk lipids. Milk exosomes.	[30,36,44,48]	Bovine skim milk (colostrum and mature milk).	[52,55]
miR-223	Neutrophil proliferation and activation. Granulopoiesis.	[118,119,120]	Skim milk. Milk lipids. Milk exosomes.	[36,44,48]	Bovine skim milk (colostrum and mature milk). Rat milk whey.	[52,54,56]
miR-150	B and T cells. Suppresses B cell differentiation.	[121,122]	Skim milk. Milk lipids. Milk exosomes.	[30,36,44,48]	Bovine skim milk (colostrum and mature milk). Rat milk whey.	[52,56]
miR-30b	Promotes induced cellular invasion. Immune suppression.	[123]	Skim milk (colostrum and mature milk). Milk lipid. Milk exosomes.	[30,36,44]	Bovine skim milk (colostrum and mature milk). Rat milk whey. Porcine milk exosomes.	[51,52,56]
miR-182	Promotes IL-2 (interleukin-2). Induces T cell-mediated immune responses.	[124]	Milk lipids. Milk exosomes.	[36,44]	Bovine skim milk (colostrum and mature milk). Rat milk whey. Porcine milk exosomes.	[51,52,56]
miR-200a	Associated with Hodgkin lymphoma.	[125]	Skim milk (colostrum and mature milk). Milk lipids. Milk exosomes.	[30,36,44]	Bovine skim milk (colostrum and mature milk). Rat milk whey.Porcine milk exosomes.	[51,52,54,56]
miR-29a	Suppresses immune responses to intracellular pathogens. Downregulated in B cell chronic lymphocytic leukemia.	[126,127]	Skim milk (colostrum and mature milk). Milk lipids. Milk exosomes.	[30,36,44]	Bovine skim milk (colostrum and mature milk). Rat milk whey. Porcine milk exosomes.	[52,54,55,56]
miR-15a	Downregulated in chronic lymphocytic leukemia.	[128,129]	Skim milk (colostrum and mature milk). Milk lipid. Milk exosomes.	[30,36,44]	Bovine skim milk (colostrum and mature milk). Porcine milk exosomes.	[52,54,55]
miR-16	Induces TNF mRNA degradation. Upregulated in rheumatoid arthritis.	[20,130]	Skim milk. Milk lipids.	[30,44]	Bovine skim milk (colostrum and mature milk). Rat milk whey. Porcine milk exosomes.	[52,55,56]
miR-21	Up-regulated in B-cell lymphoma and chronic lymphocytic leukemia.	[131]	Skim milk (colostrum and mature milk). Milk lipids. Milk exosomes.	[30,36,44]	Bovine skim milk (colostrum and mature milk). Rat milk whey. Porcine milk exosomes.	[51,52,55,56]
miR-20a	Inhibits monocyte proliferation, differentiation and maturation.	[112]	Skim milk (colostrum and mature milk). Milk lipids. Milk exosomes.	[30,36,44]	Bovine skim milk (colostrum and mature milk). Rat milk whey.	[52,54,56]
miR-106a	Inhibits monocyte proliferation, differentiation and maturation.	[112]	Milk lipids. Milk exosomes.	[36,44]	Bovine skim milk (colostrum and mature milk). Porcine milk exosomes.	[52,54,55]

In addition, microRNA clusters miR-17 and miR-92 have been detected at high levels in HM, and given their function in regulating monocyte development as well as B and T cell differentiation and maturation [18,132], they are also thought to contribute to the maturation of the infant’s immune system early in life. miR-223, which is predicted to activate proliferation of granulocytes [119], is also found at high levels in HM [48]. HM is rich in B cell-related microRNAs, such as miR-181 and miR-155, which potentially induce B cell differentiation [108,110]. On the other hand, miR-150, which is present in lower concentrations in HM, is known to act as a B cell suppressor [121,122]. Interestingly, Zhou and colleagues identified a large number of microRNAs in HM exosomes [36]. Of the 10 most abundant, 4 microRNAs were associated with immune functions, including miR-148a-3p, miR-30b-5p, miR- 182-5p, and miR-200a-3p [36]. Specifically, miR-30b-5p is known to induce immunosuppression and reduce immune cell activation [123]. In contrast, miR-182-5p induces T cell-mediated immune responses [124]. In the same study, 59 pre-microRNAs out of 87 (67.8%) that were detected in HM exosomes are considered to have immunological functions [36], which is consistent with a previous study in human skim milk microRNA [48]. The miR-17-92 cluster was also highly expressed in HM exosomes, with a speculated function as a developmental regulator of the immune system [19].

Some microRNAs present in milk may have more than one function. Interestingly, miR-17-92, which is known to have immunological functions, has also been implicated in oncogenesis by promoting cell proliferation and inhibiting apoptosis [133], although its role in the breastfed infant is poorly understood. Given that genes known to act as oncogenes have recently also been implicated in normal lactation [58,134,135], it can be hypothesised that miRN-17-92, as well as other microRNAs with similar functions may participate in the milk-secretory function of the lactating breast rather than act as oncogenes in the context of lactation and breastfeeding. Some of these microRNAs therefore may be indicators of lactation performance.

In addition to HM, microRNAs with immunological functions have been identified in the milk of other mammalian species [51,52,53,54]. Profiling of bovine milk showed a high similarity of microRNA content to HM in respect to immune-related microRNAs [48,52,53], although this does not directly translate to the human infant. miR-181a and miR-155, which play important roles in immune system regulation and inflammation [44,136], were profiled in both bovine colostrum and mature milk, and were detected in high quantities, more so in colostrum [52,53]. More specifically, bovine immune-related microRNAs are present at higher concentrations in colostrum compared to mature milk [52], although this is yet to be investigated in HM. This appears to be one of the factors involved in providing greater immunological support required early in life, and is consistent with the higher numbers of immune cells and concentrations of various humoral immunological factors such as lactoferrin and secretory IgA in colostrum compared to mature HM [58,62,137,138,139]. Bovine milk miR-15b, miR-27b, miR-34a, miR-106b, miR-130a, miR-155, and miR-223, which are all considered as immune- and development-related microRNAs, have been found in higher levels in colostrum than in mature milk [54]. Additionally, the expression levels of a selected bovine microRNA group, including miR-223, miR-106b, miR-15b, miR-155, and miR-34a, have been analyzed using qPCR and compared between colostrum and mature milk, where they were found to be present at significantly different levels [54]. In contrast to bovine milk [52,53,54], rat skim milk [56] and porcine milk exosomes [51], a study showed that the levels of microRNA concentration and expression in HM are lower in colostrum compared to mature milk, where 429 different microRNAs were identified in human mature milk *vs.* 386 different microRNAs in human colostrum [30]. This warrants validation, together with investigation of differences in immune-related microRNAs between human colostrum and mature HM, since these molecules are likely to contribute in the immunoprotection of the neonate in the first days postpartum when it is most susceptible, as well as in the development of infant’s immune system and long-term protection against infections.

Kosaka *et al.* [48] and Gu *et al.* [51] first showed that human skim milk and porcine milk exosomes, respectively, contain microRNAs related to immune responses (Table 2). Gu *et al.* found that 58 out of 84 immune-related microRNAs listed in the Pathway Central Database (Qiagen, Valencia, CA, USA) were enriched in porcine milk exosomes [51], consistent with another recent study showing that HM exosomes were enriched with immune-related microRNA [36]. This study identified 12 out of 13 high abundance microRNAs in porcine milk exosomes to be expressed at higher levels in the first 3 days postpartum compared to later in month 1 postpartum (days 7, 14, 21 and 28) [51]. These 12 microRNAs (let-7a-5p, miR-182-5p, miR-191-5p, miR-200c-3p, miR-21-5p, miR-25-3p, miR27b-3p, miR-30a-5p, miR-30c-2-5p & -1-5p, miR-30d-5p, miR-375-3p, and miR-574-3p) [51] are all immune-related and they regulate immune response genes and proteins [140]. More specifically, miR-30c-2-5p and miR-1-5p are immunosuppression regulators [123], whereas let-7a-1-5p regulates inflammation-associated cytokine IL-6 (interleukin-6) that induces STAT3 (signal transducers and activators of transcription 3) signaling [141]. The innate immune receptors can be activated and regulated by porcine milk miR-21-5p, toll-like receptor 4 (TLR4), and a key cytokine receptor via targeting programmed cell death protein 4 (PDCD4) and interleukin 12 (IL-12), respectively [142]. Also, IL-12 that is negatively controlled by miR-21-5p, is also responsible for regulating T cells and natural killer cells [143]. Further, the abundant porcine milk miR-27b was found to induce lipopolysaccharide (LPS), which inhibits and de-stabilizes the peroxisome proliferator-activated receptor c (PPARc), which is important in dampening inflammation via macrophage immune response [144].

Several bovine immune-related microRNAs [100,101] have been isolated from milk-derived microvesicles [53], and were also found to be expressed in the mammary gland using small RNA sequencing (Table 2) [145]. These microRNAs include miR101 and miR150, which are known regulators of T cells [146,147], and also miR-223 that has been reported to modulate innate immune cell (granulocytes and neutrophils) differentiation and activation [100,119,120]. miR-155 and miR-223 have been detected in bovine milk and are both involved in many immune functions [100], and potentially have anti-inflammatory effects especially in bovine colostrum. In addition to this function, miR-155 regulates T and B cell differentiation, and is a known modulator of T helper cells (Th1/Th2 balance) [148]. In contrast, miR-223 negatively regulates neutrophil proliferation and activation [100]. miR-25-3p targets KLF4, which is a potent mediator of inflammation [149], and has a crucial role in the development of the immune system [51]. miR-30a-5p targets GalNActransferase 7 (GALNT7) to promote cellular invasion and immunosuppression [123]. miR-182-5p promotes T cell-mediated immune responses by inhibiting forkhead box protein O1 (FOXO1) [124], a gene that is also targeted by miR-21 in cancer [150,151]. miR-200c-3p has been identified to regulate T cell differentiation by targeting zinc finger E-box-binding homeobox 1 (ZEB1) [152], and also to regulate CD4 differentiation [153]. Moreover, as mentioned earlier, bovine milk microRNA expression patterns have been recently found to be altered during severe inflammation of mammary gland such as mastitis [71]. Gene target analysis of the up- and down-regulated milk microRNAs such as miR-223 and miR-15b, respectively, revealed several roles of these microRNAs in response to mastitis [154]. Also, most milk microRNAs were downregulated during mastitis, suggesting that they actively control the mammary immune response to *S. uberis,* which causes mastitis in the dairy cow [154].

Collectively, the current data highlight that breastmilk is a complex system of different microRNA molecules with synergistic and antagonistic relationships, controlling specific immune responses in the infant and the lactating breast [36,42,44,48]. Factors such as the stage of lactation (colostrum *vs.* mature milk) and infection/inflammation have been shown to influence the microRNA-mediated epigenetic regulation of immune responses and development in both the infant and the lactating breast, further supporting the potential use of these molecules diagnostically.

### 3.3. microRNAs Are Key Regulators of Milk Lipid Metabolism

MicroRNAs have been isolated from HM lipid vesicles including fat globules in large numbers [44], as well as from milk cells and skim milk [44,46,155]. This has formed the basis for the potential use of both extracellular and intra-vesicle milk microRNAs as biomarkers in molecular diagnostics for a range of diseases [156]. Although lipid metabolism is usually regulated extracellularly, microRNAs have recently been identified to regulate genes associated with lipid metabolism at the post-transcriptional level [155]. These genes control functions related to cholesterol homeostasis, fatty acid oxidation, and lipogenesis, offering new opportunities for the treatment of various diseases such as dyslipidemias [157].

The known lipid regulatory microRNAs are few and include amongst others miR-335, miR-33, miR-122, miR-370, miR-378-3p, and miR-125a-5p [157]. Interestingly, these microRNAs have been identified in abundance in the HM lipid fraction [44], human skim milk [30], HM cells [35], human colostrum [30], HM exosomes [36], as well as in bovine skim milk and colostrum [52], suggesting that they play critical roles in the lipid metabolism and/or synthesis in the lactating breast. For example, miR-33 has been shown to regulate cholesterol homeostasis at the cellular level [158,159]. One of the most significant predicted gene targets of miR-33 is ABCA1, which produces cholesterol efflux regulatory protein (CERP). CERP is responsible for regulating cellular cholesterol and phosphate homeostasis, and also transporting cholesterol outside of the cell [160]. miR-33 also targets ABCG1, which reduces the efflux of cholesterol to high-density lipoprotein (HDL) and serum in macrophages [156,161].

miR-125a-5p is another microRNA found abundantly in human and other species milk, regulating oxysterol binding protein-related Protein 9 (ORP9) [162], which is involved in various processes of lipid metabolism [163,164] including induction of lipid uptake by macrophages [162]. Furthermore, HM miR-103 [30,44] is known to regulate milk fat synthesis, promoting fat globule synthesis and accumulation of triglyceride and unsaturated fatty acids [165]. Overexpression of miR-103 has been identified as a crucial regulator of milk fat synthesis and composition as well as milk nutrient levels [165]. Interestingly, downregulation of miR-103 was not shown to affect fat accumulation in caprine milk lactocytes, suggesting that there may be alternative and/or compensatory mechanisms controlling mammary fat metabolism [165]. Further, miR-193b and miR-365, also present in milk, were shown to control lipid synthesis, upregulating brown fat differentiation via enhancing expression of Runt-related transcription factor 1 translocated to 1 (Runx1t1) [166].

Interestingly, in addition to fat globule-related microRNA, many microRNA have been found to be packaged into other lipid-based carriers, such as exosomes, microvesicles and apoptotic bodies, which are secreted by various cell types, such as immune cells [167], and many of which are found in HM [36]. These are known as lipid particle carriers [168,169], packaging not only microRNA, but also lipoproteins [155], and have the important function of delivering extracellular microRNA to recipient cells. For example, miR-150 is transported via microvesicles from macrophage-like cells to human microvascular endothelial cells, where it is thought to target c-Myb and regulate cell migration [122,170,171]. Moreover, adipocyte-derived microRNA such as miR-27a, miR-146b and miR-16, are transported to other cell recipients via microvesicles [171]. Therefore, it can be postulated that microRNAs contained in milk microvesicles/exosomes as well as fat globules are transferred to recipient cells in the GI tract of infants, and a proportion may transfer to the blood circulation from where they are transported to the infant’s tissues, playing regulatory functions.

### 3.4. Various Potential Benefits of Human Milk microRNAs

The function of extracellular microRNA is still poorly understood [48]. Current evidence supports the notion that extracellular microRNAs play crucial roles in cell-cell communication [42,172,173]. microRNAs have been shown to be exported by cells in culture [42]. Moreover, proteins and mRNA can be taken up by neurons through exosomes from adjacent cells, suggesting that the same is possible for microRNA [42]. The existence of microRNA in exosomes and their potential function as extracellular regulators have opened up a new field of possibilities for use of microRNAs as biomarkers in health and disease [86,88] as well as in therapeutic modeling [174,175].

HM microRNA are potentially involved in many physiopathological functions, including regulating cell growth and differentiation [48] as well as influencing development in the infant [52]. For example, one of the most highly expressed microRNA in HM, miR-148a-3p [35,44], which is also found in other species’ milk [51,52], targets DNA methyltransferase 3b (DNMT3B) and suppresses its expression, potentially to facilitate DNA methylation during development [176]. At the same time, given that the majority of cells in mature HM under healthy conditions are lactocytes [58], HM microRNA are reflective of the microRNA composition and function of the lactating mammary epithelium, and this can form the basis for further explorations of their use as non-invasive, easily accessible biomarker of the functionality of the lactating breast. 

Moreover, some tissue-related microRNAs have been found in HM [48], but less abundantly than in tissue and organs [36]. For example, miR-142-5p and miR-142-3p (hematopoietic system), miR-122 (liver), and miR-216 and miR-217 (pancreas) were highly expressed in these organs and less abundantly in HM [48,177], suggesting that these HM microRNAs may originate from the maternal bloodstream to specifically target the development, growth and function of the corresponding organs in the HM fed infant. At the same time, they may have specialized functions in the breast during lactation. Similarly in bovine milk, microRNA have been identified as tissue-specific microRNA present in low quantities in milk and with lower expression in both bovine colostrum and mature milk [52]. These include for example muscle miR-1 and miR-133 [173], brain miR-9 and miR-124a [178], pancreatic miR-216 and miR-217 [179], liver miR-122 [21,173], blood cell miR-451 [180], and endothelial cell miR-126 [181].

microRNAs isolated from HM fat globules have been shown to be regulated by a maternal high-fat diet, and this may modify metabolic pathways in HM fed infants [44]. This is in agreement with the putative roles of circulating microRNAs, which when altered in either composition or concentration can be associated with cardiovascular morbidity and mortality [182,183]. Munch *et al.* found that gene targets of 308 microRNAs in HM lipids have a wide range of functions, particularly in the regulation of gene expression and metabolism, and immune responses [44], suggesting the potential importance of these microRNAs for HM fed infants [44,184]. Further to these functions, some HM microRNA are thought to participate in the regulation of the central nervous system (CNS). For example, Munch *et al.* [44] showed that HM miR-118.2 targets Teneurin Transmembrane Protein 2 (TENM2), the encoding protein of which is found at high levels in the CNS [185], suggesting a regulatory function in the infant’s neural development and promotion of connection formation within the nervous system [186,187]. Adipogenesis may also be targeted in the infant via milk-derived microRNAs. Overexpression of miR-155 [54] has been speculated to decrease brown adipose tissue mass by targeting the adipogenic transcription factor CCAAT/enhancer-binding protein β (C/EBPβ) [188]. Milk-derived miR-29a inactivates the INSIG-1 gene [30,44,52], which is likely to regulate adipogenesis [189], and this was positively associated with body max index (BMI) [190]. In addition to direct effects on fat deposition, milk microRNA may be involved in the short- and/or long-term appetite control conferred to the infant via breastfeeding, together with the numerous appetite regulatory hormones of breastmilk, such as leptin, adiponectin, ghrelin, insulin and others [191].

In addition to their involvement in normal metabolism and tissue function, many microRNAs have been shown to target genes related to cancer [192,193], with some of these gene targets known to increase or decrease cancer risk. These microRNAs could be used as cancer biomarkers for both prognosis and diagnosis [43] and some of them are present in milk, and more specifically in HM. Although epidemiological evidence has previously associated bovine milk consumption with increased risk of certain cancers in adults [194,195,196,197,198,199], and this could be related to the content of bovine milk in oncogenic microRNA [200], the microRNAs in HM appear to have normal lactation-specific functions for the lactating mammary gland and the infant [45]. Interestingly, HM microRNA have been proposed to protect the infant against cancer through to adulthood [44]. For example, miR-21, which is present in both HM and bovine milk [36,52], is also known to be overexpressed in human hepatocellular cancer (HCC). Therefore, any deregulation of miR-21 can be associated with HCC growth by modulating mTORC1 signaling, *i.e.*, PTEN expression [201]. miR-21 is an abundant microRNA in bovine milk [52] and has been isolated from both colostrum and mature HM [30]. It is also abundant in human plasma [202], and in infants it is thought to be involved in promoting postnatal growth [203]. In addition, miR-21 has other normal tissue functions, including regulation of adipogenic differentiation in mesenchymal stem cells (MSCs) of human adipose tissue [204]. Further, HM microRNAs may directly regulate tumor suppressor genes [205], such as the *let-7* family, which is involved in decreasing lung tumor growth by directly targeting the *RAS* oncogene [206]. The specific normal functions of HM microRNA for the infant and in the lactating breast warrant further investigation.

## 4. Infant Formula is Poor in microRNAs Compared to Human Milk

HM is much more than nutrition for the infant, containing fat, carbohydrates, proteins, vitamins and minerals, but also immunoprotetive and regulatory biomolecules as well as viable cells that provide essential signals for the infant’s optimal growth, development and protection [70,191,207,208,209]. However, a rapid worldwide population growth over the last 100 years, and the high demand to provide artificial milk for infants has led scientists and industrial companies to successfully produce infant formula from bovine milk as an alternative or complementary food for infants initially without access to HM [210]. And although many have expressed the view that infant formula should only be made available to the infant if mother’s own milk is not sufficient, infant formula has recently become controversially more popular within some communities for non-medical reasons [211].

HM is a complex biofluid containing maternal somatic cells, beneficial microbiota, and molecules including microRNAs with functional roles [30,134,212,213,214]. Most infant formulae are bovine milk-based [44], and similar to HM, bovine milk microRNAs are most likely to be highly conserved in both fat globules (lipid fraction) and cells. Due to their stability, microRNAs may largely survive the industrial milk preparation procedures, however the milk cell and lipid fractions are usually discarded from formula [215,216], so the microRNA presence in formulae is significantly reduced. This has been confirmed by studies showing that the expression level of microRNAs in formulae is much lower than that of raw bovine milk (Table 3) [52,54,217]. Chen *et al.* selected and surveyed seven microRNAs as quality control markers for raw milk and different brands of infant formula [52]. The expression level of these seven microRNAs was significantly lower in formulae compared to raw milk [52]. Izumi *et al.* found that the total RNA concentration of three types of infant formulae (standard formula, follow-on formula, and extensively hydrolyzed formula) was significantly lower than in raw bovine milk [54]. Also, two highly expressed microRNAs (miR-148a and miR-200c) in bovine milk were differentially expressed among the three different types of infant formula [54]. In a recent study, we have compared the human microRNA content and expression levels of two infant formulae from the Australian market, a bovine-milk based and a soy-based formula [46]. Out of 754 human mature microRNAs tested using Taqman Openarray (Applied Biosystems), only 45 microRNAs were identified in the bovine milk formula, and only 22 microRNAs in the soy formula [46], (Table 3). Moreover, the biological activity of the remaining few formula microRNAs may be altered by the formula processing procedures, something that requires further investigation. Although the functional effects of non-human milk microRNAs on infants have not been investigated, it is possible that some microRNAs that are shared between bovine and human milk may play similar beneficial functions for the offspring across mammals, emphasizing the potential detrimental effects for infants of the low microRNA content of artificial formulae. This, together with the near absence in formulae of other immunoprotective factors of HM are likely to at least partially explain the reduction in protection from disease in infants fed artificial formulae [217]. Donor milk is the preferred alternative to formula, and even if pasteurized, donor milk is likely to retain many HM microRNA, potentially conferring more benefits to the infant than formula.

**Table 3:** Comparison of selected microRNAs and their abundance in infant formulae, bovine milk, and human milk (HM).

MicroRNA	Mature Sequence (miRBase 20.0)	Existence	Expression Level	References
bta-miR-26a hsa-miR-26a-5p	UUCAAGUAAUCCAGGAUAGGCU UUCAAGUAAUCCAGGAUAGGCU	Bovine milk. Infant formula. HM.	Low in formulae compared to raw bovine milk. Highly expressed in HM lipid fraction.	[44,52]
bta-miR-26b hsa-miR-26b-5p	UUCAAGUAAUUCAGGAUAGGUU UUCAAGUAAUUCAGGAUAGGU	Bovine milk. Infant formula. HM.	Low in formulae compared to raw bovine milk. Highly expressed in HM lipids and exosomes.	[36,44,52]
bta-miR-200c hsa-miR-200c-3p	UAAUACUGCCGGGUAAUGAUGGA UAAUACUGCCGGGUAAUGAUGGA	Bovine milk. Infant formula. HM.	Low in formulae compared to raw bovine milk. Highly expressed in HM lipid fraction.	[44,52,54]
bta-miR-21-5p hsa-miR-21-5p	UAGCUUAUCAGACUGAUGUUGACU UAGCUUAUCAGACUGAUGUUGA	Bovine milk. Infant formula. HM.	Low in formulae compared to raw bovine milk. Highly expressed in HM lipids, skim milk and exosomes.	[30,36,44,48,52]
bta-miR-30d hsa-miR-30d-5p	UGUAAACAUCCCCGACUGGAAGCU UGUAAACAUCCCCGACUGGAAG	Bovine milk. Infant formula. HM.	Low in formulae compared to raw bovine milk. Highly expressed in HM lipids and skim milk.	[30,44,52]
bta-miR-99a-5p hsa-miR-99a-5p	AACCCGUAGAUCCGAUCUUGU AACCCGUAGAUCCGAUCUUGUG	Bovine milk. Infant formula. HM.	Low in formulae compared to raw bovine milk. Highly expressed in HM lipids and skim milk.	[30,44,52]
bta-miR-148 hsa-miR-148a-3p	UCAGUGCACUACAGAACUUUGU UCAGUGCACUACAGAACUUUGU	Bovine milk. Infant formula. HM.	Low in formula compared to raw bovine milk. Highly expressed in HM lipid, skim milk and exosomes.	[30,36,44,52,54]

Notes: hsa refers to human, bta refers to *Bos taurus* (bovine) species.

## 5. Conclusions and Outlook

microRNAs play beneficial functions in humans and are actively involved in many normal developmental and physiological processes. They are crucial modulators of many normal functions, such as cardiac function and other cardiovascular processes, immune protection, and tissue function [218]. Deregulation of microRNAs has also been shown to be associated with disease, for which they are useful diagnostic biomarkers [192,219]. The recent discovery and identification of microRNA in HM requires further study to elucidate the biology of microRNA and their normal functions in the development and protection of the human breast and the infant. Although the use of circulating microRNA as biomarkers is still in its infancy [156], microRNA have been proposed as biomarkers for various abnormalities [86], including breast cancer [220,221] and more recently, milk microRNA as biomarkers for lactation performance [52] and mastitis [71]. This is of particular interest since milk can be accessed easily and non-invasively and is plentiful. Importantly, microRNAs are extremely stable, and are transferred to humans via food, and also to infants via HM. Infant formula not only contains insufficient amounts of biologically active microRNAs, but it also has a completely different microRNA profile to human milk, with potential detrimental effects on the growth, development and protection of the infant. The investigation of the roles of HM microRNA for the infant and the mother will not only reveal novel attributes of breastfeeding, but it may also open new diagnostic and therapeutic avenues.
